# Improved Model for Starch Prediction in Potato by the Fusion of Near-Infrared Spectral and Textural Data

**DOI:** 10.3390/foods11193133

**Published:** 2022-10-08

**Authors:** Fuxiang Wang, Chunguang Wang

**Affiliations:** Mechanical and Electrical Engineering, Inner Mongolia Agricultural University, Hohhot 010018, China

**Keywords:** hyperspectral imaging, starch content, potato, data fusion, partial least squares regression

## Abstract

In this study, visible-near-infrared (VIS-NIR) hyperspectral imaging was combined with a data fusion strategy for the nondestructive assessment of the starch content in intact potatoes. Spectral and textural data were extracted from hyperspectral images and transformed principal component (PC) images, respectively, and a partial least squares regression (PLSR) prediction model was then established. The results revealed that low-level data fusion could not improve accuracy in predicting starch content. Therefore, to improve prediction accuracy, key variables were selected from the spectral and textural data through competitive adaptive reweighted sampling (CARS) and correlation analysis, respectively, and mid-level data fusion was performed. With a residual predictive deviation (RPD) value > 2, the established PLSR model achieved satisfactory prediction accuracy. Therefore, this study demonstrated that appropriate data fusion can effectively improve the prediction accuracy for starch content and thus aid the sorting of potato starch content in the production line.

## 1. Introduction

After wheat, rice, and maize, potato ranks as the fourth largest vegetable crop worldwide. According to the data of the Food and Agriculture Organization of the United Nations, global potato production in 2019 reached 36.91 million tons. Potato tubers are rich in starch, proteins, amino acids, and many vitamins and minerals [1]. Starch content is a critical indicator of potato quality because it not only determines the nutritional value of potatoes but also contributes to their taste quality [2]. Furthermore, potato starch can be used in meat products, pasta, and confectionery products because of its high viscosity, transparency, and propensity for gelling. Therefore, it is very important to develop an accurate method to measure the starch content in potatoes.

Conventionally, the starch content in potatoes is determined through chemical analysis in a laboratory. However, this method takes a lot of time and is not ecofriendly because the process damages the sample and requires tedious sample pretreatment [3]. Therefore, new nondestructive testing methods for the rapid determination of potato starch content are urgently required. Hyperspectral imaging (HSI) is a rapidly developing and green technology that has the abilities of near-infrared spectroscopy (NIRS) and computer vision techniques. HSI can not only acquire spectral information of the tested sample, but also capture the external features of the sample, including its shape and texture. Therefore, HSI has been used in numerous studies for analyzing the quality of potatoes, including their physical properties, such as the color changes in potato tubers during processing [4], and textural properties, such as the potato’s hardness, stickiness, and extent of binding during consumption [5]. Researchers have used HSI to determine the internal components of potatoes, including starch and cellulose [6] and glucose, fructose, and sucrose [7]. However, the aforementioned studies have focused only on spectral information in hyperspectral images but have not considered spatial image information. Some studies have revealed that the textural information in hyperspectral images can be used to predict the physical properties and chemical components of potatoes. For example, Khulal et al. (2016) assessed the content of total volatile basic nitrogen in chicken using HSI. Their results revealed the feasibility of using textural data in the quantitative prediction of total volatile basic nitrogen [8]. Wang et al. (2020) used spectral data and quality indicators to predict and visualize palmitic acid in mutton. Their model outperformed the single-spectrum data model. Furthermore [9], Zhang et al. (2020) revealed that combining spectral and textural features can make HSI more accurate in predicting the contents of fat and moisture in salmon [10]. However, the feasibility of predicting starch content by using textural data in hyperspectral images has not been comprehensively investigated. At present, no researchers have combined spectral and texture characteristics to predict the starch content in potatoes.

Thus, this study aimed to investigate the ability of combining HSI with chemometric modeling to predict the starch content in potatoes. Specifically, our objectives were to: (1) collect the near-infrared-hyperspectral images of intact potato samples of various varieties; (2) extract the spectral and textural data from hyperspectral images; (3) develop prediction models that are based on spectral/textural data; and (4) compare predictive performance for starch content when single versus multiple types of information are used.

## 2. Materials and Methods

### 2.1. Sample Preparation

In this study, potato samples were purchased from a supermarket in Hohhot, Inner Mongolia. Two widely grown local potato varieties, namely Kexin No.1 and Holland No.15, were selected for the study. Mature and fresh potatoes with no surface defects were selected. In total, 96 potatoes consisting of 68 Kexin No.1 and 28 Holland No.15 potatoes were selected. The surfaces of all potatoes were immediately washed with water in the laboratory. The potatoes were dried with absorbent paper and numbered. Furthermore, the potatoes were kept away from light for 24 h.

### 2.2. Hyperspectral Images Collection of Potato Samples

The hyperspectral images of the potato samples were all collected using a benchtop visible-near-infrared (VIS-NIR) hyperspectral imager, which consists of an imaging spectrometer (modelIV10E, Spectral Imaging, Oulu, Finland), a lens with a focal length of 23 mm (Imperx, Boca Raton, FL, USA), a charged-coupled device camera with a resolution of 1600 × 1200 pixels (IGV-B1620, Imperx, Boca Raton, FL, USA), having two tungsten halogen lamps (3900, Illumination Technologies, NY, USA) and a stepper motor-controlled moving platform (IRCP0076-1 COM, Taiwan, China). A line scan working in the reflection mode was used in this system. The hyperspectral imager was first switched on for 30 min. The acquired spectra were in the range 382–1004 nm and comprised 428 consecutive wavelengths. The original hyperspectral images were then corrected according to the method described in [11].

### 2.3. Spectral Data Extraction from Hyperspectral Images

The hyperspectral images after correction were imported into ENVI software (ITT Visual Information Solutions, Boulder, CO, USA), and the spectral data were averaged within the region of interest (ROI) of 100 × 100 pixels. Totally, 96 spectra of 96 potato samples were obtained. The raw spectra of the potato sample contained instrument noise and chemical information. Therefore, spectral preprocessing was performed on the raw spectral data to remove the noise. After referring to some previous studies and a preliminary comparison of different preprocessing methods, we used the standard normal variate (SNV) method for preprocessing the raw spectra in this study. The advantage of SNV was described in [12].

### 2.4. Textural Data Extraction

The obtained hyperspectral images contained continuous grayscale images at 428 wavelengths, producing a redundant, computationally intensive block of three-dimensional data. Therefore, dimensionality reduction of the hyperspectral data was necessary, for which we used principal component analysis (PCA). PCA is a classic data dimensionality reduction algorithm [13], and it was used to transform the original 428-dimensional grayscale image to the first 3-dimensional PC images, namely the PC1, PC2, and PC3 images. For each sample, three PC images were collected; thus, a total of 96 × 3 = 288 PC images were obtained.

The PC images of the potato samples contained crucial textural features that could be related to the starch content. Therefore, these textural features should be extracted. The Gray level co-occurrence matrix (GLCM) [14], a widely used textural feature extraction method, was used in this study to extract textural features. Four commonly used textural features, namely correlation, contrast, entropy, and uniformity, were extracted by using the GLCM [15]. Here, the selected PC images were used to extract textural features in four directions (0°, 45°, 90°, and 135°) at a distance of 1. Subsequently, the averaged values of the four directions were used as the representative textural features [16]. Thus, a total of 12 textural features were obtained for each potato sample.

### 2.5. Chemical Analysis of the Starch Content in the Potato Samples

The starch content of each potato sample was determined through enzymatic hydrolysis as described in [17]. All measurements were taken in triplicate. Subsequently, the average of the three measurements was taken as the starch content of that sample.

### 2.6. Data Fusion Strategy

Data fusion was used to enhance the synergy of the fused information by using complementary data inputs to achieve superior prediction performance. In general, the data fusion strategies that are commonly used are either low-level or mid-level fusion strategies [18]. In low-level fusion, all data sources are simply stitched together into a matrix. Low-level fusion can retain the maximum information about the data. However, some irrelevant variables are also retained, which may lead to the prediction of redundant tasks and a less accurate prediction model. In addition, all data were used for modeling, which resulted in a large quantity of data for modeling and long computation times [18,19]. In mid-level fusion, numerous relevant features are first extracted from each data source separately. These features are then joined into a single array for further multivariate regression. This approach not only extracts data that are relevant to the target compound, but also markedly simplifies the modeling process. In this study, low- and mid-level fusion strategies were used to merge the spectral and textural data. For low-level fusion, all 96 × 428 spectral variables and 96 × 12 textural variables were fused, and a new matrix of 96 × 440 variables were obtained. For mid-level fusion, the raw data from the spectra and textures were first subjected to competitive adaptive reweighted sampling (CARS) and correlation analysis, respectively, to select the characteristic variables within them. These selected variables were then fused to form a new data matrix.

### 2.7. Establishment of the Quantitative Model

In this study, the correlation between hyperspectral data (spectra, textures, fusion of spectra, and textures) and the target starch content was established by partial least squares regression (PLSR) [20,21]. In the PLSR model, matrices X (hyperspectral data) and Y (starch content) can be considered simultaneously. Furthermore, the model can be used for dealing with a large of variables, including collinear variables, in the raw data. The raw data were transformed into independent latent variables (LVs) using PLSR. In this study, the maximum number of LVs was set to 15, and a five-fold cross-validation method was used to obtain the optimal number of LVs [21]. The correlation coefficients of the calibration set (Rc) and the prediction set (Rp), the RMSE of the calibration set (RMSEC) and prediction set (RMSEP), and the residual predictive deviation (RPD) were used to evaluate the prediction performance of the PLSR models. A more accurate prediction model has higher Rc, Rp, and RPD values and lower RMSEC and RMSEP values. All modeling procedures were performed in MATLAB. 

## 3. Results and Discussion

### 3.1. Measured Starch Content in the Potato Samples

The statistical results of the measured starch content in potato samples are presented in Table 1. Potato samples of two varieties, namely Kexin No.1 and Holland No.15, which are widely grown in Inner Mongolia, were analyzed. For Kexin No.1, the starch content ranged from 1.77 to 18.31 g/100 g, with an average of 10.23 ± 2.95 (expressed as mean ± standard deviation) g/100 g. For Holland No.15, the starch content ranged from 17.01 to 22.81 g/100 g, with an average of 18.96 ± 1.29 g/100 g. Furthermore, five-fold internal cross-validation was used to optimize the model parameters (the number of LVs) during the modeling process, which prevented under- or overfitting of the model. In this study, all potato samples were sorted according to starch content, after which the middle sample of every three samples was grouped into the prediction set and the remaining two into the calibration set. Thus, 64 and 32 samples were present in the calibration and prediction sets, respectively. The statistical results of the starch content in the two sample sets are presented in Table 1. The starch content in the calibration and prediction sets were in the ranges of 1.77–22.81 and 5.04–21.01 g/100 g, respectively. After sample division, the span of the starch content in the prediction set was covered by that in the calibration set, which facilitated the establishment of robust prediction models.

### 3.2. Spectral Characteristics of Potato Samples

**Figure 1:**
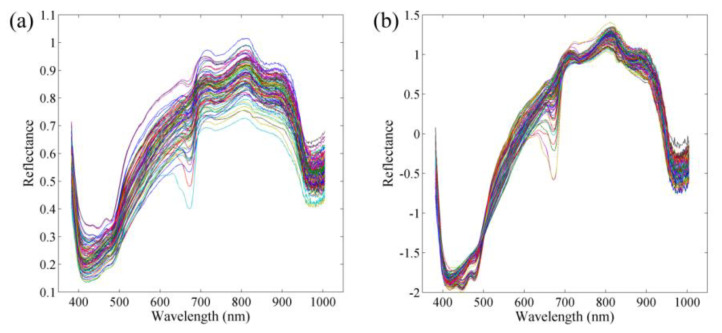
(**a**) Raw; (**b**) SNV preprocessed spectral curves of all potato samples.

The raw spectral curves of all potato samples are displayed in Figure 1a, where three clear absorption peaks are present at approximately 410, 680, and 980 nm. The absorption peak at 410 nm was probably due to the absorption of carbohydrates, whereas the absorption peak at 680 nm was due to the fourth C–H stretching overtone band of glucose and fructose [22,23]. The apparent absorption peak at 980 nm was due to the presence of water molecules [23] Furthermore, absorption peaks were observed at 750 and 850 nm, which were associated with stretching vibrations in the C–H and O–H bands in this region [24]. Considerable burr-like noise in the raw spectral curves was observed, which may adversely affect the modeling process and ultimately reduce model accuracy. Therefore, the SNV approach was used to preprocess the raw spectra, and the results are displayed in Figure 1b. After preprocessing, the noise was somewhat reduced, but the position of these distinct absorption peaks did not change.

### 3.3. Quantitative Model Based on a Single Type of Data

The PLSR models of the starch content were established by using the raw spectra, SNV pretreated spectral, and textural data, and the results are displayed in Table 2. For the raw spectral data, the PLSR model achieved satisfactory results with an Rc of 0.9052 and an RMSEC of 2.03 g/100 g for the calibration set. However, when the prediction set was used to validate the PLSR model, a lower prediction accuracy was achieved, with an Rp of 0.8102, RMSEP of 2.81 g/100 g, and RPD of 1.67. According to the criteria provided by Williams [25], an RPD value under 2.0 indicates the unsuitability of a method for quality assessment, and an RPD value over 2.0 indicates that it can be applied for rough screening. Thus, the PLSR model based on raw spectral data failed to accurately predict the starch content in potato samples. After SNV preprocessing, the PLSR model performed better than the model based on raw data, with a higher Rp value of 0.8584, a higher RPD value of 1.89, and a lower RMSEP value of 2.48 g/100 g. However, it was still difficult to accurately predict the starch content using the PLSR model based on preprocessed spectral data.

**Figure 2:**
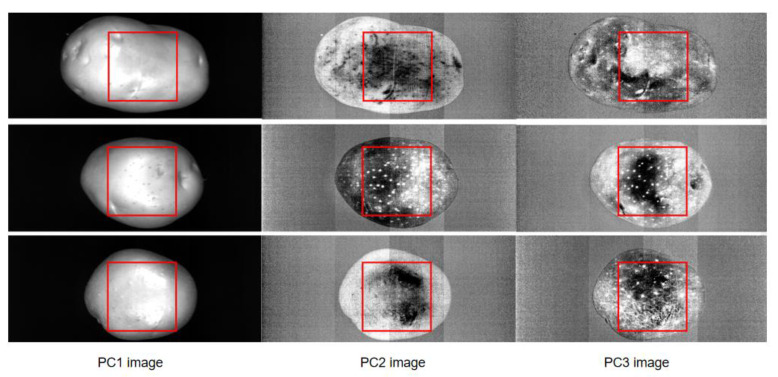
PC images extracted from the hyperspectral images of potato samples.

To extract textural data from hyperspectral images, the high-dimensional data blocks were transformed using PCA for dimensionality reduction, the first three PC images (PC1, PC2, and PC3 images) were used for textural data extraction, as displayed in Figure 2. For textural data, the PLSR model yielded poor performance in starch content prediction, with an Rc of 0.5451, RMSEC of 4.02 g/100 g, Rp of 0.7271, RMSEP of 3.29 g/100 g, and RPD of 1.43 (Table 2). The accuracy of the prediction model based on textural data was lower than that of the model based on spectral data, which indicated that starch content had a considerably higher correlation with spectral information than with textural information.

### 3.4. Quantitative Model Based on Fused Data

**Figure 3:**
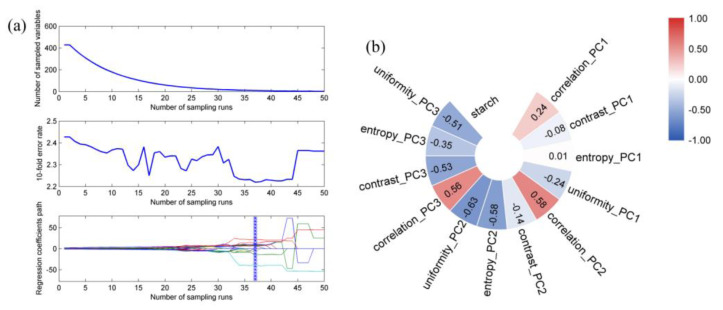
(**a**) Results of CARS method for wavelength selection; (**b**) correlation plot between textural variables and measured starch content.

The aforementioned results indicate that PLSR models based on one type of data (either spectral or textural data) cannot accurately predict starch content, as indicated by their RPD values being lower than 2.0. Therefore, in this section, the two types of data were fused to establish the PLSR model for predicting starch content. Low-level and mid-level data fusion strategies were introduced, and the developed PLSR model results are displayed in Table 2. For low-level data fusion, the 428 variables from the spectral matrix and the 12 variables from the textural matrix were combined to produce a new matrix containing 440 variables. The PLSR model achieved poor predictive performance in starch content prediction, with an Rc of 0.8606, RMSEC of 2.44 g/100 g, Rp of 0.8467, RMSEP of 2.53 g/100 g, and RPD of 1.86. Although data fusion results in larger data sets, in this study, data fusion did not considerably improve the model’s predictive performance. Low-level fusion simply overlays all the data, including irrelevant and redundant data, which may reduce the accuracy of the model. Therefore, mid-level data fusion was used. In our mid-level data fusion, the key variables from the spectra were selected using a CARS method, whereas key variables from the textures that correlated strongly with starch content were selected using a correlation analysis method. The selection results are presented in Figure 3. From the findings in Figure 3a, we extracted 10 important wavelengths (478, 479, 481, 482, 828, 907, 909, 915, 934, and 944 nm) from the full spectra, which indicated that 97.66% of the total wavelengths were eliminated using CARS. As indicated in Figure 3b, various textural variables exhibited different correlations with the starch content. Specifically, the absolute values of the correlation coefficients between seven variables and the starch content were higher than 0.3, with uniformity_PC2 exhibiting the largest negative correlation with a correlation coefficient of −0.63, and correlation_PC2 exhibiting the largest positive correlation with a correlation coefficient of 0.58. Thus, these seven textural variables were selected as key variables and used with the ten wavelengths for mid-level data fusion. The PLSR model based on data subject to mid-level fusion provided acceptable performance, with an Rc of 0.8911, an RMSEC of 2.17 g/100 g, an Rp of 0.8832, an RMSEP of 2.29 g/100 g, and an RPD of 2.05. The RPD value was higher than 2.0, which indicates that the proposed PLSR model can be used for the accurate prediction of the starch content in potato samples. Compared with low-level data fusion, mid-level fusion not only markedly improved the prediction accuracy of the model, but also considerably reduced the number of variables used, thereby simplifying the modeling process. In recent years, there have not been much research on the detection of potato starch content by hyperspectral imaging technology. Jiang [26] established a least square regression prediction model by using hyperspectral equipment, smoothing as pretreatment and principal component analysis as feature extraction, and predicted the starch content of potatoes with an Rp of 0.9031 and a RMSEP of 0.5025. This model has good predictability, but the sample of this model was the same potato variety, and its adaptability to other varieties is unknown. At present, no one has studied the effect of information fusion on starch content of potato varieties.

## 4. Conclusions

Vis-NIR HSI was combined with PLSR modeling to predict the starch content in intact potato samples. Spectral and textural data were extracted from the ROI of hyperspectral images and transformed PC images, respectively. Our results revealed that neither frontal spectrum nor textural data alone can be used to accurately predict starch content. After low-level data fusion, the performance of the established PLSR model did not greatly improve. However, mid-level data fusion resulted in satisfactory prediction performance when applied in a PLSR model, as indicated by an RPD value greater than 2.0. In conclusion, our study established a method for evaluating the starch content of various varieties of intact potatoes and aiding the industrial grading of potatoes.

## Figures and Tables

**Table 1:** Statistical results of measured starch content (g 100 g^−1^) in potato samples of two varieties.

Dataset	Number	Min	Max	Mean	SD
Kexin No.1	68	1.77	18.31	10.23	2.95
Holland No.15	28	17.01	22.81	18.96	1.29
Calibration set	64	1.77	22.81	12.78	4.80
Prediction set	32	5.04	21.01	12.77	4.71

SD: standard deviation.

**Table 2:** Performance of PLSR models for starch content prediction by using different data.

Data	NVs	LVs	Calibration Set		Prediction Set		
			Rc	RMSEC	Rp	RMSEP	RPD
Raw spectra	428	5	0.9052	2.03	0.8102	2.81	1.67
SNV preprocessed	428	5	0.8719	2.34	0.8584	2.48	1.89
Texture	12	3	0.5451	4.02	0.7271	3.29	1.43
Low-level fusion	440	5	0.8606	2.44	0.8467	2.53	1.86
Mid-level fusion	17	5	0.8911	2.17	0.8832	2.29	2.05

NVs: number of variables; LVs: number of latent variables; Rc: correlation coefficient in calibration set; RMSEC: root mean square error in calibration set; Rp: correlation coefficient in prediction set; RMSEP: root mean square error in prediction set; RPD: residual predictive deviation.
